# Establishment and Characterization of a Brain-Derived Cell Line from Japanese Eel (*Anguilla Japonica*) and Transcriptomic Analysis of Its Responses to Sex Steroid Hormones

**DOI:** 10.3390/ani16182951

**Published:** 2026-09-19

**Authors:** Yajuan Huang, Xu Yan, Songlin Chen

**Affiliations:** 1 State Key Laboratory of Mariculture Biobreeding and Sustainable Goods, Yellow Sea Fisheries Research Institute, Chinese Academy of Fishery Sciences, Qingdao 266071, China; yjhuang230622@163.com (Y.H.);; 2 College of Life Science, Qingdao University, Qingdao 266071, China

**Keywords:** *Anguilla japonica*, brain-derived cell line, steroid hormones, transcriptome

## Abstract

The Japanese eel (*Anguilla japonica*) is an economically important aquaculture species, but cellular mechanisms underlying sex steroid responses remain poorly characterized. In this study, we established and characterized a continuous brain-derived cell line from Japanese eel, designated AJBC. The cells exhibited stable fibroblast−like morphology and continuous proliferation. Molecular characterization showed high expression of *col1a1*, a marker associated with fibroblast−like stromal populations, whereas neuronal and glial markers (*map2*, *gfap*, and *sox2*) showed low expression. The neural-associated marker *nestin* was detectable, indicating that AJBC cells retained certain brain-associated characteristics. AJBC cells were successfully transfected with a GFP−expressing plasmid, demonstrating their potential for genetic manipulation. Transcriptome analysis identified 3212 and 3522 differentially expressed genes following 17β−estradiol (E2) and 11-ketotestosterone (11−KT) treatment, respectively. KEGG analysis revealed enrichment of PI3K−Akt and MAPK signaling pathways, as well as cell adhesion and cytoskeletal-related processes. Representative reproductive-related genes, including *sox9* and *foxl2*, showed differential expression following sex steroid hormone treatment. E2 increased *esr1* and *esr2* expression, whereas 11−KT increased *ar* expression, confirming the steroid-responsive characteristics of AJBC cells. This brain-derived cell line provides a useful in vitro model for investigating sex steroid−responsive molecular mechanisms and neuroendocrine-related processes in Japanese eel.

## Simple Summary

Japanese eel (*Anguilla japonica*) is an important aquaculture species, but the biological processes underlying its reproduction and sex development are not yet fully understood. The brain plays a key role in regulating reproductive processes, yet suitable in vitro models for studying eel brain cells are limited. In this study, we established a stable brain-derived cell line from Japanese eel that could be continuously maintained under laboratory conditions. The cells showed stable growth and characteristics suitable for laboratory research, including the capacity for exogenous gene introduction. Exposure to sex steroid hormones caused substantial changes in gene expression, particularly in processes related to cell communication, cell structure, and metabolism. These findings indicate that the established cell line can respond to sex steroid hormones and provides a useful in vitro model for studying brain–endocrine regulation and reproduction in Japanese eel.

## 1. Introduction

The Japanese eel (*Anguilla japonica*) is an economically important aquaculture species in East Asia, particularly in China, Japan, and Korea, where recruiting glass eels are extensively collected and cultured for food production [1]. However, Japanese eel populations and recruitment have declined markedly over recent decades, with habitat loss, overfishing, migration barriers, and environmental and oceanographic changes considered important contributing factors [2,3]. Accordingly, A. japonica was classified as Endangered (EN) on the IUCN Red List in 2014, highlighting the need for effective conservation and sustainable aquaculture practices [4]. Although substantial progress has been made in artificial maturation, induced spawning, and larval rearing, commercially viable closed-cycle production remains challenging [5,6]. A major bottleneck is the incomplete understanding of the endocrine and molecular mechanisms governing reproductive maturation and development, which limits further optimization of artificial reproduction technologies [7].

In teleosts, reproductive development is controlled primarily through the hypothalamic–pituitary–gonadal (HPG) axis, in which the brain functions as the central regulatory organ by integrating environmental, nutritional, and endocrine cues and coordinating reproductive endocrine activities [8,9]. Neural circuits within the brain integrate GnRH, kisspeptin, dopamine, and other neuroendocrine signals to modulate pituitary gonadotropin release and gonadal steroidogenesis [10]. In addition to regulating endocrine activity, the brain is also an important target organ of circulating steroid hormones. Estrogens and androgens act on neural cells through genomic and non-genomic signaling pathways and regulate neuronal function, synaptic plasticity, neurogenesis, and reproductive behavior [11,12,13]. Increasing evidence suggests that interactions between steroid hormones and the central nervous system play essential roles in the regulation of reproductive function in teleost fishes [8,14]. Therefore, elucidating the direct responses of brain cells to sex steroid hormones is important for understanding the neuroendocrine mechanisms underlying reproduction and hormone-responsive processes in Japanese eel [15].

Among the major sex steroids in teleosts, 17β−estradiol (E2) and 11-ketotestosterone (11−KT) are key regulators of female and male reproductive processes, respectively [14]. Sex steroid signaling also influences neuroendocrine regulation within the brain and brain–pituitary–gonadal axis, thereby contributing to reproductive development and function [8]. The expression of steroid hormone receptors and downstream-responsive genes in the brain enables neural cells to respond directly to circulating sex steroids, providing an important basis for investigating steroid-responsive cellular mechanisms. Recent transcriptomic studies in teleosts have further demonstrated that exogenous sex steroid exposure can alter gene expression and regulatory networks in the brain during reproductive processes [16]. However, most previous studies have relied on whole tissue analyses or in vivo hormone administration, making it difficult to distinguish direct cellular responses from secondary systemic endocrine effects [14]. Therefore, the lack of a brain-derived in vitro model represents an important limitation for investigating the direct molecular responses of Japanese eel brain cells to sex steroid hormones.

The establishment of stable fish cell lines provides reproducible and experimentally tractable in vitro models for studying cellular physiology, gene function, signaling pathways, host–pathogen interactions, and toxicological responses [17]. Compared with primary cultures, stable cell lines can be maintained over extended periods and provide greater experimental consistency, facilitating repeated molecular and functional analyses [18]. To date, cell lines have been established from a wide range of teleost tissues, including gonad, liver, kidney, skin, muscle, and brain, and have been extensively applied in studies of fish physiology, immunology, virology, toxicology, and biotechnology [19,20,21,22]. In Japanese eel, cell lines derived from tissues such as kidney and spleen have also been established, providing useful in vitro systems for investigating cellular physiology and host–pathogen interactions [23]. Brain-derived cell lines have also been established in several teleost species, providing useful in vitro models for investigating neural function, viral infection, toxicological responses, and neuroendocrine regulation [22,24,25,26]. However, no stable brain-derived cell line from Japanese eel (*Anguilla japonica*) has been reported to date, limiting cellular and molecular investigations of steroid-responsive neuroendocrine regulation in this species.

In the present study, we established a stable brain--derived cell line, designated AJBC, from Japanese eel (*Anguilla japonica*) using the tissue explant adhesion method. The biological characteristics of AJBC cells, including cell morphology, growth characteristics, chromosome stability, species identification, and transfection efficiency, were systematically characterized. Furthermore, the transcriptional responses of AJBC cells to E2 and 11−KT treatment were investigated using RNA sequencing, followed by differential expression and functional enrichment analyses to identify hormone-responsive genes and pathways associated with neuroendocrine and reproductive processes. This study establishes a novel in vitro cellular platform for investigating steroid hormone responses in the Japanese eel brain. It provides a useful model for studying neuroendocrine signaling and hormone-responsive cellular mechanisms in *A. japonica*.

## 2. Materials and Methods

### 2.1. Experimental Animals

Healthy Japanese eels (*Anguilla japonica*; approximately 450 g in body weight and 65 cm in total length) were obtained from a commercial aquaculture farm in Fuqing, Fujian Province, China, in May 2025. A single fish was used for cell line establishment, and one brain tissue sample from this individual was used to generate the primary cell culture. Before tissue collection, the fish was maintained in an aerated freshwater tank under laboratory conditions and fasted for 24 h. Following anesthesia with 100 mg/L tricaine methanesulfonate (MS–222; Sigma-Aldrich, St. Louis, MO, USA), the brain and gonadal tissues were collected aseptically from the same donor fish at the same time. The brain tissue was used for primary cell culture, while the gonadal tissue was subjected to histological examination, which indicated that the donor fish was female at the mid stage of ovarian development. All experimental procedures were performed in accordance with the Guidelines for the Care and Use of Laboratory Animals of the Yellow Sea Fisheries Research Institute, Chinese Academy of Fishery Sciences, and were approved by the Institutional Animal Care and Use Committee (Approval No. YSFRI-2025017).

### 2.2. Primary Cell Culture and Subculture

Brain tissues were aseptically collected from healthy Japanese eels (*Anguilla japonica*) and minced into approximately 1 mm^3^ fragments. After repeated washing with PBS containing 1% penicillin-streptomycin, the tissue fragments were treated with 0.25% trypsin-EDTA for 15 min and then placed onto T25 culture flasks using the tissue explant adhesion method. The cultures were maintained at 27 ± 2 °C, with half of the culture medium replaced on days 2 and 3 and complete replacement on day 4. Thereafter, the medium was changed every 2–3 days. When the cells reached 80–90% confluence, they were detached with 0.25% trypsin-EDTA and subcultured at a ratio of 1:2–1:3. The subculture medium consisted of L-15 medium supplemented with 10% FBS, 1% penicillin−streptomycin, 20 ng/mL FGF, 10 ng/mL EGF, and 50 µM β−mercaptoethanol. Subsequently, the cells were passaged every 2–3 days.

### 2.3. Cryopreservation and Recovery

AJBC cells at approximately 80–90% confluence in T25 culture flasks were harvested and digested with 0.25% trypsin-EDTA for 1 min, followed by low-speed centrifugation at 800 rpm for 5 min to collect the cell pellet. The cells were gently resuspended in freezing medium consisting of 90% fetal bovine serum (FBS; Gibco, Grand Island, NY, USA) and 10% DMSO and mixed thoroughly to ensure a uniform cell suspension. The cell suspension was then aliquoted into cryovials and maintained at 4 °C for 1 h before being transferred to a controlled-rate freezing container. The cells were cooled at approximately 1 °C/min to −80 °C and stored overnight, followed by transfer to liquid nitrogen for long-term storage.

To assess cell recovery viability, the cryovials were rapidly thawed in a 37 °C water bath with gentle agitation until the ice was completely melted. The thawed cells were immediately centrifuged at 1000 rpm for 5 min. After removal of the supernatant, the cell pellet was gently resuspended in culture medium. Cell viability was assessed by trypan blue exclusion using an inverted microscope, with unstained cells considered viable and blue-stained cells considered non-viable. Cell viability was calculated as the percentage of viable cells among the total number of counted cells.

### 2.4. COI Gene Sequencing and Comparative Analysis

AJBC cells from a T25 culture flask, which had been continuously cultured for approximately 100 days and reached passage 30, were collected, and total RNA was extracted using the TRIzol method. cDNA was synthesized using a reverse transcription kit (Vazyme, Nanjing, China). The mitochondrial cytochrome oxidase subunit I (COI) gene (GenBank: PP514564.1) was amplified by PCR using specific primers for Japanese eel: COI-F, 5′−GGTACCCTATATCTAGTATTTGGTG−3′; and COI-R, 5′−TGGGTGACCAAAGAATCAGAATAG−3′. The 50 μL PCR reaction mixture contained 25 μL of TaKaRa 2xTaq, 2 μL of cDNA template, 19 μL of H_2_O, and 2 μL each of COI-F and COI-R primers (10 μM). PCR amplification was performed with an initial denaturation at 95 °C for 5 min, followed by 38 cycles of denaturation at 95 °C for 30 s, annealing at 58 °C for 30 s, and extension at 72 °C for 1 min, with a final extension at 72 °C for 5 min. The PCR products were analyzed by 1% agarose gel electrophoresis, and the target bands were purified and submitted to Sangon Biotech (Qingdao, China) for sequencing. The obtained sequences were compared and analyzed against sequences in the NCBI database using BLAST web tool (https://blast.ncbi.nlm.nih.gov/Blast.cgi accessed on 15 September 2025) to confirm the identity of the AJBC cell line.

### 2.5. Determination of the Optimal Culture Conditions

To determine the optimal FBS concentration for AJBC cell culture, healthy cells at passage 30 were digested with 0.25% trypsin–EDTA to prepare a single-cell suspension. The cell density was determined using an automated cell counter and adjusted to 1 × 10^4^ cells/mL. The cells were seeded into 12-well culture plates at 1 mL per well, with three independent biological replicates for each treatment, and cultured at 27 °C for 24 h to allow sufficient cell attachment. After attachment, the culture medium was replaced with L–15 medium supplemented with 5%, 10%, 15%, or 20% FBS and 1% penicillin-streptomycin, and the cells were subsequently maintained at 27 °C. Cells were collected on days 0,1, 2, 3, 4, 5, 6, and 7. After washing once with PBS, the cells were detached with 0.25% trypsin, and digestion was terminated by adding culture medium. The cells were resuspended to obtain a single–cell suspension, and viable cell numbers were determined using an automated cell counter. Growth curves were generated based on the viable cell numbers at each time point to evaluate the effects of different FBS concentrations on AJBC cell growth.

To determine the optimal culture temperature for AJBC cells, passage 30 cells were digested with 0.25% trypsin-EDTA to prepare a single−cell suspension, and the cell density was adjusted to 1 × 10^4^ cells/mL using an automated cell counter. The cells were seeded into 12-well culture plates at 1 mL per well, with three independent biological replicates for each treatment. After sufficient cell attachment, the cells were cultured at 20, 24, 27, or 30 °C under otherwise identical culture conditions. Cells were collected on days 0, 1, 2, 3, 4, 5, 6, and 7, and viable cell numbers were determined using an automated cell counter following trypsinization. Growth curves were generated based on the viable cell numbers at each time point to evaluate the effects of different culture temperatures on AJBC cell proliferation.

### 2.6. Chromosome Analysis

For karyotype analysis, cells from two confluent T25 culture flasks were subcultured at a ratio of 1:2 into four T25 flasks and cultured until 70–80% confluence. Colchicine was then added to the culture medium at a final concentration of 1 μg/mL, and the cells were incubated for 6 h. The cells were subsequently harvested by digestion with 0.25% trypsin-EDTA and subjected to hypotonic treatment with 4 mL of 0.075 M KCl for 30 min. The cells were then pre−fixed with 4 mL of ice−cold Carnoy’s fixative (methanol: glacial acetic acid = 3:1) for 3 min. After centrifugation, the supernatant was discarded, and the cells were fixed again with 4 mL of freshly prepared Carnoy’s fixative for 20 min. The fixation step was repeated 1–2 times. After centrifugation at 1000 rpm for 10 min, the cell pellet was gently resuspended in a small volume of Carnoy’s fixative and dropped onto glass slides pre-cooled with absolute ethanol at −20 °C from a height of approximately 1 m. The slides were briefly heated over an alcohol lamp to facilitate chromosome spreading and examined under a microscope to assess cell density and chromosome dispersion. Slides with appropriate chromosome spreading were subsequently prepared using the same procedure. The chromosomes were stained with Giemsa solution for 40 min, gently rinsed with distilled water, and air-dried at room temperature. Metaphase chromosome spreads were examined and photographed under a microscope, and chromosome numbers were counted in 100 well−spread cells.

### 2.7. Cell Transfection and GFP Expression

AJBC cells from one confluent T25 culture flask were seeded into one six−well culture plate and cultured at 27 °C until the cell density reached approximately 80%. Lipo8000^TM^ transfection reagent, serum-free culture medium, and the pEGFP-N1 plasmid were mixed and incubated according to the manufacturer’s instructions, and the mixture was subsequently added to the cells. After 48 h of culture, GFP fluorescence was observed and photographed using an inverted fluorescence microscope. Transfection efficiency was quantified by calculating the percentage of GFP-positive cells among total cells in randomly selected fields.

### 2.8. Molecular Characterization of AJBC Cells

To characterize the molecular features of the AJBC cell line, the expression profiles of neural associated marker genes were analyzed. Total RNA was extracted from AJBC cells and Japanese eel brain tissues using TRIzol reagent (Invitrogen, USA) according to the manufacturer’s instructions. The extracted RNA was reverse-transcribed into cDNA for PCR amplification. The expression levels of neural associated marker genes, including sex-determining region Y-box 2 (*sox2*), *nestin*, microtubule-associated protein 2 (*map2*), glial fibrillary acidic protein (*gfap*), and collagen type I alpha 1 chain (*col1a1*), were examined to characterize the cellular properties of AJBC cells. Gene–specific primers were used for quantitative PCR amplification, and β−actin was used as an internal control. Relative expression levels were calculated using the 2^−ΔΔCt^ method. Three independent biological replicates were performed for each group. Primer sequences used in this study are listed in Table 1.

### 2.9. E2 and 11−KT Hormone Treatment of AJBC Cells

AJBC cells in good growth condition with approximately 80% confluence were digested with trypsin, resuspended in complete culture medium containing 20% FBS, and adjusted to a density of 1 × 10^4^ cells/mL. The cell suspension was seeded into 96-well plates at 100 μL per well and incubated overnight at 27 °C. The medium was then replaced with freshly prepared complete culture medium containing 0, 10, 100, or 1000 nM E2 or 11−KT. E2 and 11−KT were dissolved in DMSO, and the control group received an equivalent concentration of DMSO as the vehicle control. Cells were subsequently cultured at 27 °C, and cell viability was assessed at 12, 24, 36, and 48 h using the CCK-8 assay. Three independent biological replicates were included for each treatment group. Briefly, 10 μL of CCK–8 solution was added to each well and incubated at 27 °C for 1–2 h. The absorbance at 450 nm (OD450) was measured using a microplate reader, and cell viability was calculated based on the OD450 values and compared among the different treatment groups. Based on the CCK–8 assay results, appropriate hormone concentration and treatment duration were selected for subsequent experiments. The same serum concentration was maintained during hormone treatment to avoid additional stress caused by serum withdrawal and ensure consistent culture conditions.

### 2.10. Expression Analysis of Sex Steroid Hormone Receptors in AJBC Cells

To evaluate the potential responsiveness of AJBC cells to sex steroid hormones, the expression patterns of sex steroid hormone receptor genes were analyzed following E2 and 11−KT treatment. AJBC cells were treated with E2 or 11−KT under the selected experimental conditions, and the control group received an equivalent concentration of DMSO as the vehicle control. Total RNA was extracted and reverse-transcribed into cDNA. The expression levels of estrogen receptor genes (*esr1* and *esr2*) and the androgen receptor gene (*ar*) were determined by quantitative real–-time PCR (qPCR). β-actin was used as the internal reference gene, with three independent biological replicates included for each group. Relative expression levels were calculated using the 2^−ΔΔCt^ method. Primer sequences used in this study are listed in Table 1.

### 2.11. Transcriptome Analysis of Sex Hormone-Treated AJBC Cells

Based on preliminary experiments, the optimal hormone concentration and treatment duration were determined to be 100 nM and 24 h, respectively. AJBC cells at passages 30 were seeded into 6--well culture plates and cultured to approximately 80% confluence. The culture medium was then replaced with fresh Leibovitz’s L-15 medium supplemented with 20% FBS containing 100 nM E2 or 100 nM 11−KT. Cells cultured in the same medium without hormone supplementation served as the control group. Each treatment was performed with three independent biological replicates. Following 24 h of hormone treatment, cells were collected, immediately frozen in liquid nitrogen, and stored at−80 °C until RNA extraction.

Total RNA was extracted from AJBC cells using TRIzol^TM^ Reagent (Invitrogen, Waltham, MA, USA), and RNA quality and integrity were assessed using a NanoDrop 2000 spectrophotometer (Thermo Scientific, Wilmington, DE, USA). Qualified RNA samples were used for transcriptome library construction. Total mRNA was enriched using mRNA Capture Beads, fragmented, and reverse-transcribed into double-stranded cDNA. The cDNA libraries were subsequently subjected to end repair, A-tailing, adapter ligation, and PCR amplification. The constructed libraries were sequenced using the Illumina HiSeq 2500 platform by Ouyi Biology (Shanghai, China), generating 150 bp paired-end (PE150) reads. Raw reads were filtered using fastp (v0.18.0) to remove adapters, ambiguous bases, and low-quality reads, and rRNA-derived reads were discarded using Bowtie2 (v2.4.4). Qualified clean reads were mapped to the *Anguilla japonica* reference genome and corresponding annotation files (available at Figshare: https://doi.org/10.6084/m9.figshare.31151467) using HISAT2 (v2.1.0). Gene-level expression represented by fragments per kilobase of transcript per million mapped reads (FPKM) and transcripts per million (TPM) were quantified using Salmon (v1.10.3). DESeq2 (v1.24) was used for differential expression analysis of samples with biological replicates, while edgeR (v3.12.1) was applied for datasets without biological replicates. Differentially expressed genes were identified using the criteria of FDR < 0.05 and |fold change| ≥ 2. Hypergeometric test-based Gene Ontology (GO) and Kyoto Encyclopedia of Genes and Genomes (KEGG) enrichment analyses were performed with an FDR-adjusted threshold of ≤0.05. Gene expression values were subjected to row-wise z-score normalization for visualization, and heatmaps were generated using the pheatmap package (https://www.bioinformatics.com.cn/plot_basic_cluster_heatmap_plot_024 accessed on 15 September 2025).

### 2.12. Real-Time Fluorescent Quantitative PCR (qPCR) Detection

To assess the consistency of the transcriptome sequencing results, several genes were randomly selected from the identified differentially expressed genes (DEGs) and analyzed by quantitative real-time PCR (qRT-PCR). Total RNA was extracted from AJBC cells using TRIzol^TM^ Reagent (Invitrogen, Carlsbad, CA, USA) according to the manufacturer’s instructions. RNA concentration and purity were determined using a NanoDrop 2000 spectrophotometer (Thermo Fisher Scientific, Wilmington, DE, USA). First-strand cDNA was synthesized from 1 μg of total RNA using the PrimeScript^TM^ RT Reagent Kit with gDNA Eraser (Takara Bio Inc., Dalian, China). Gene sequences were downloaded from the NCBI database, and primers were designed using SnapGene software (V7.2) (Table 1).

Quantitative real-time PCR was performed using TB Green^®^ Premix Ex Taq^TM^ II (Takara Bio Inc., Dalian, China) on a QuantStudio^TM^ 5 Real-Time PCR System (Applied Biosystems, Foster City, CA, USA). The PCR amplification program consisted of an initial denaturation at 95 °C for 30 s, followed by 40 cycles of 95 °C for 5 s and 60 °C for 30 s. Melting curve analysis was performed after amplification to verify the specificity of each PCR product. The expression stability of three candidate reference genes (*18S* rRNA, *β-actin*, and *gapdh*) was evaluated in the control and hormone-treated groups, and β-actin was selected as the reference gene. The amplification efficiency of the β-actin primers was further validated. Relative expression levels were calculated using the 2^−ΔΔCt^ method. Three independent biological replicates were included for each treatment group. Statistical differences among groups were determined using one-way ANOVA followed by Tukey’s multiple comparison test, with *p* < 0.05 considered statistically significant.

## 3. Results

### 3.1. Establishment and Morphological Characterization of the AJBC Cell Line

**Figure 1:**
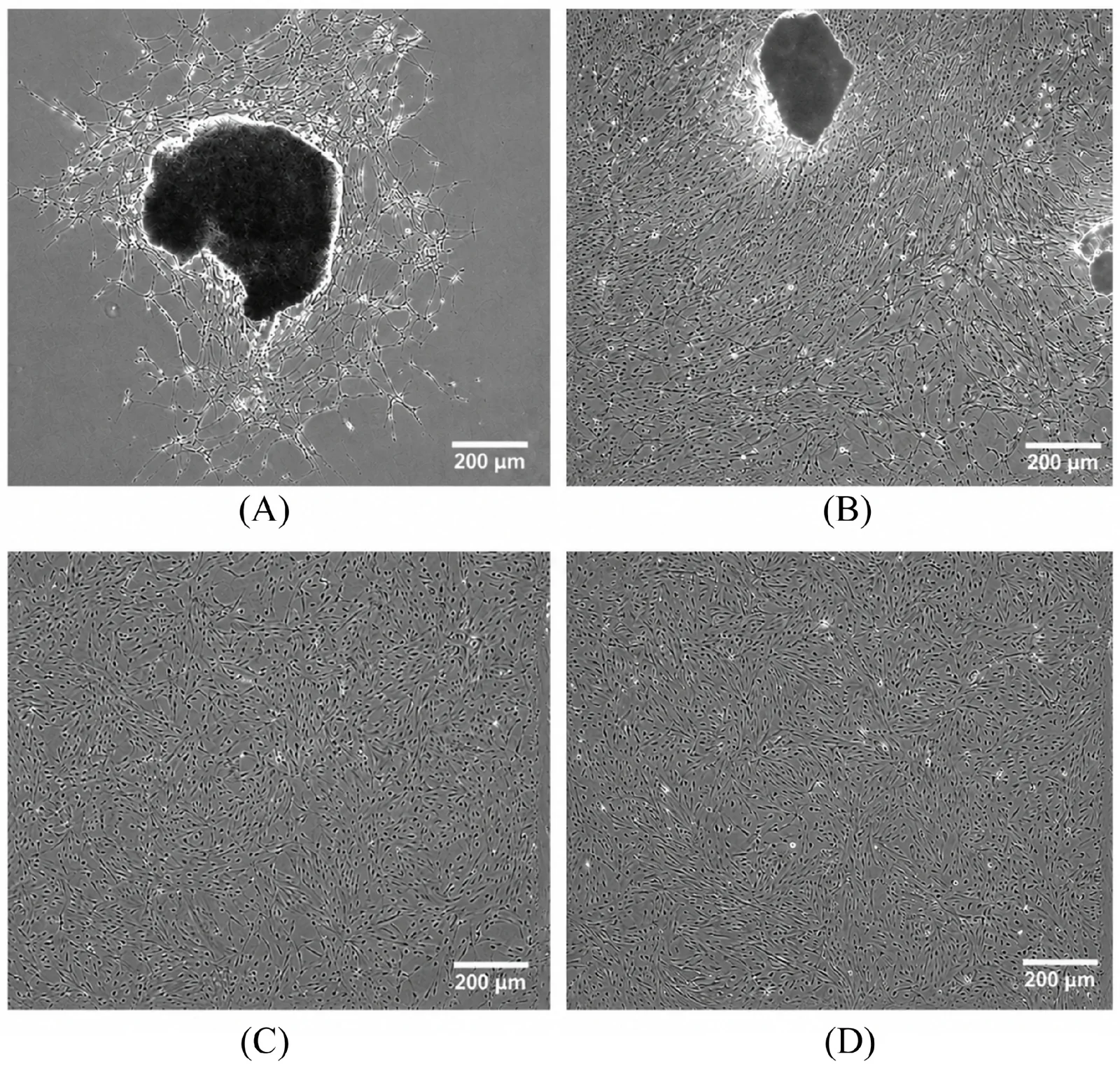
Morphological characteristics of primary and subcultured cells derived from Japanese eel (*Anguilla japonica*) brain tissue. (**A**) Primary cells cultured for 7 days; (**B**) primary cells cultured for 15 days; (**C**) AJBC cells at passage 30; (**D**) AJBC cells recovered from cryopreservation.

A primary brain cell culture was established from Japanese eel (*Anguilla japonica*) using the tissue explant culture method. Approximately 3–5 days after explant attachment, cells began to migrate radially from the edges of the tissue fragments (Figure 1A,B). The migrated cells exhibited spindle-shaped or fibroblast-like morphology and gradually proliferated to form a confluent monolayer (Figure 1C). Following repeated subculture, the cells maintained a relatively uniform morphology and continued to proliferate, and the established cell line was designated AJBC (Figure 1D). During continuous passaging, the cells maintained stable morphology and growth rates, with no evidence of mixed cell populations, abnormal swelling, vacuolization, or growth retardation. To minimize cross-contamination, cells were cultured separately using dedicated equipment, with regular medium changes and continuous morphological monitoring. All procedures were performed in a dedicated biosafety cabinet, and no morphological or growth abnormalities suggestive of contamination or mixed cell populations were observed throughout the study. The AJBC cell line was deposited at the China General Microbiological Culture Collection Center (CGMCC) on 23 April 2026 under accession number CGMCC No. 46802.

### 3.2. Growth Characteristics of the AJBC Cell Line

**Figure 2:**
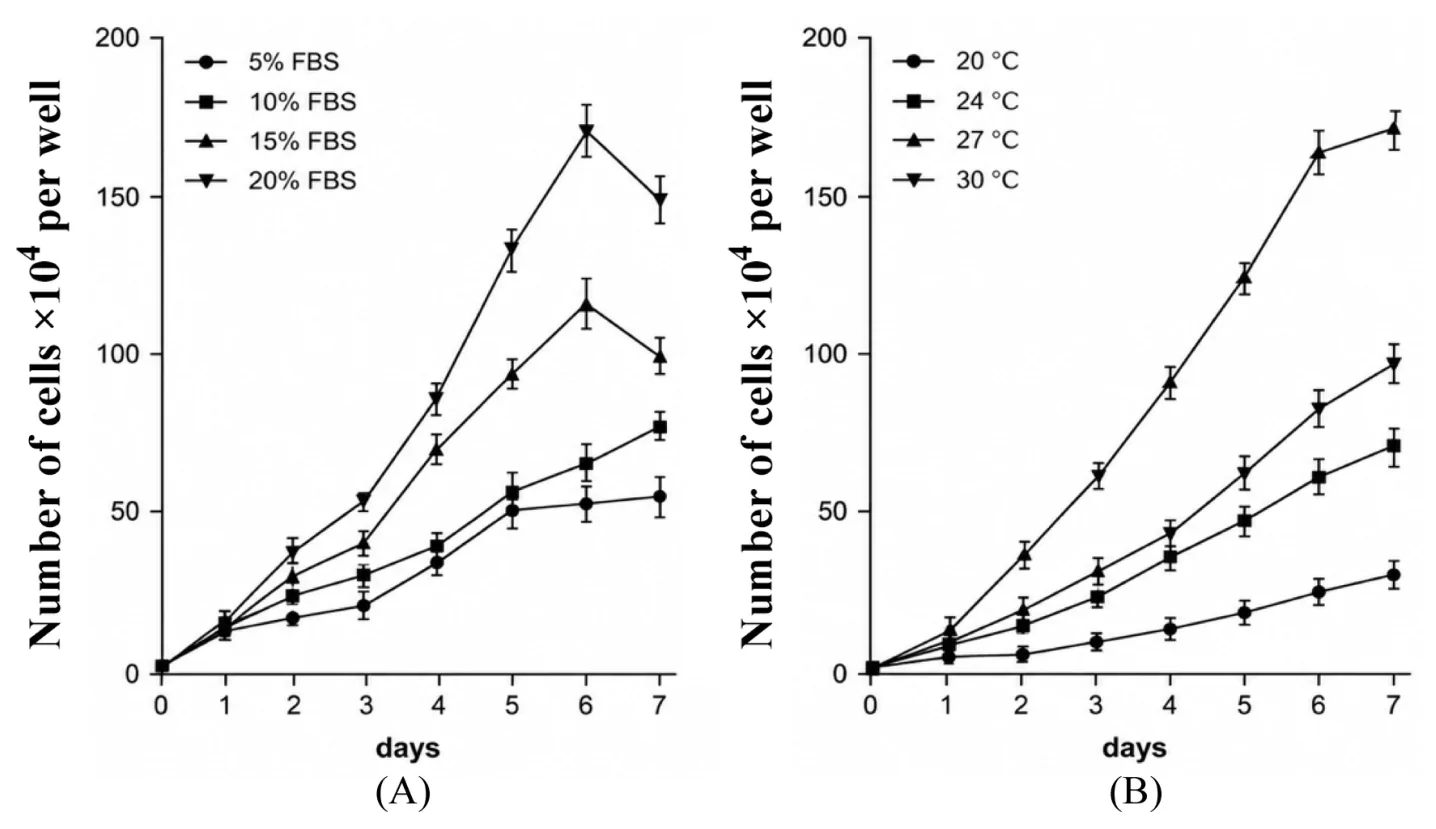
Growth characteristics of the brain-derived AJBC cell line established from Japanese eel (*Anguilla japonica*) under different culture conditions. (**A**) Growth curves of AJBC cells cultured with different concentrations of FBS; (**B**) growth curves of AJBC cells cultured at different temperatures. Data are presented as the mean ± SD from three independent biological replicates (*n* = 3). Statistical significance was determined using two–way ANOVA followed by Tukey’s multiple comparison test.

As shown in Figure 2A, AJBC cells proliferated progressively over the 7–day culture period at all FBS concentrations tested, but the growth patterns differed among groups. Cell proliferation increased more rapidly in the 15% and 20% FBS groups, particularly from days 2 to 6, with the 20% FBS group reaching the highest cell density on day 6. In contrast, cells cultured with 5% and 10% FBS showed slower proliferation. From day 6 to day 7, cell numbers decreased in the 15% and 20% FBS groups, whereas the 10% FBS group continued to increase and the 5% FBS group remained relatively stable. These results indicate that the effect of FBS concentration on cell proliferation varies with culture time.

The effects of incubation temperature on AJBC cell growth are shown in Figure 2B. Cell numbers increased over time at all tested temperatures, but the growth patterns differed among temperature groups. Cells cultured at 27 °C showed progressively faster proliferation from days 2 to 7, reaching the highest cell density on day 7. The 30 °C group also showed relatively rapid proliferation during the later culture period, whereas cells at 24 °C proliferated more steadily. In contrast, the 20 °C group showed the slowest proliferation throughout the experimental period. These results indicate that the effect of incubation temperature on cell proliferation varies with culture time. All experiments were performed with three independent biological replicates (*n* = 3), each derived from a separate cell culture experiment.

### 3.3. Cryopreservation and Recovery of the AJBC Cell Line

The cryopreservation and recovery characteristics of the AJBC cell line were evaluated to assess its suitability for long-term storage and subsequent use. Following cryopreservation in freezing medium containing 10% DMSO and serum, AJBC cells were successfully recovered after thawing. Trypan blue exclusion analysis showed that the post-thaw cell viability was consistently above 70% (Table 2).

### 3.4. Species Identification of the AJBC Cell Line

**Figure 3:**
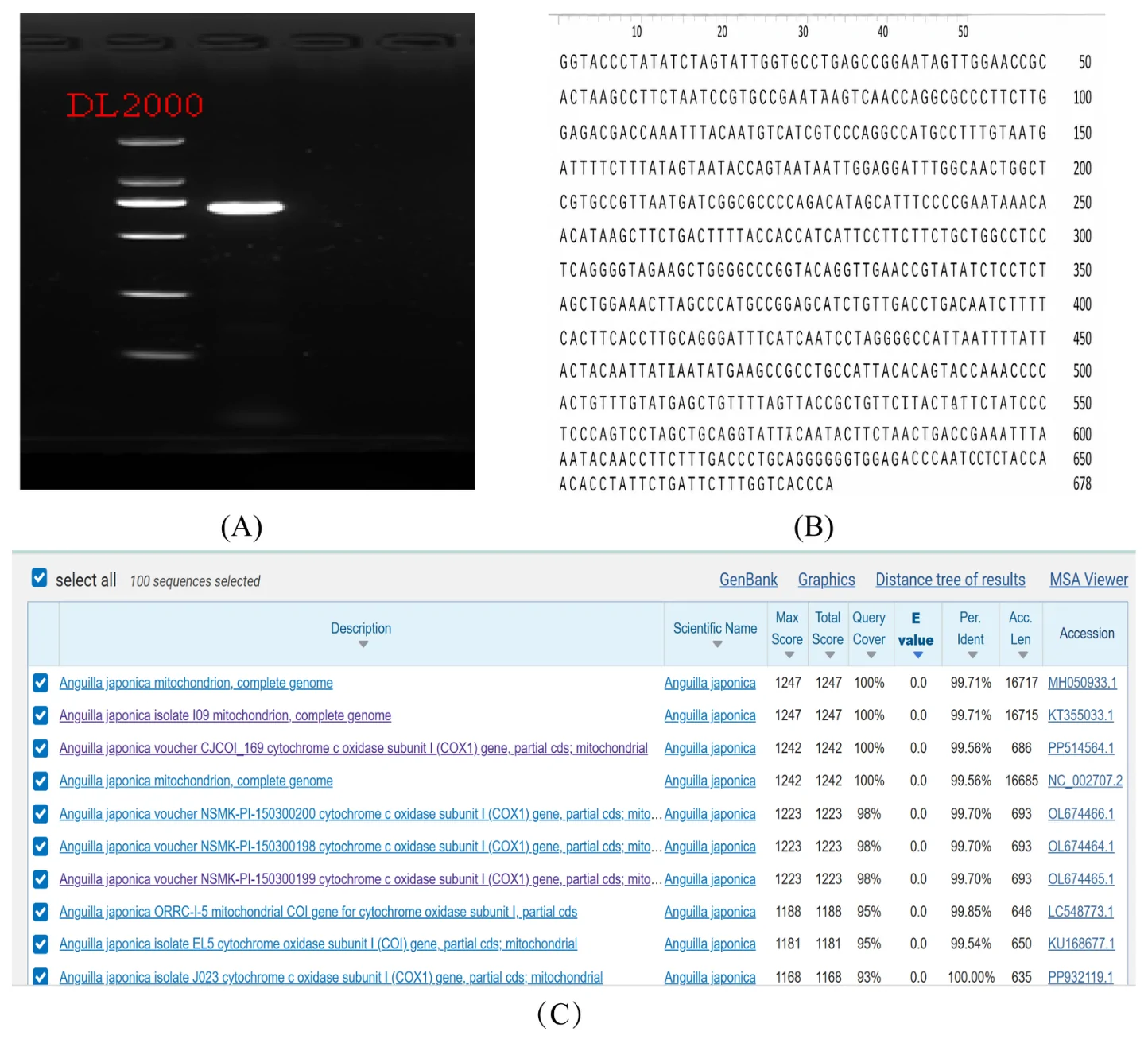
Identification of the mitochondrial cytochrome c oxidase subunit I (COI) gene sequence of the Japanese eel (*Anguilla japonica*) AJBC cell line. (**A**) Agarose gel electrophoresis of the PCR-amplified mitochondrial COI gene fragment from the AJBC cell line. (**B**) The 678bp nucleotide sequence of the mitochondrial COI gene obtained from the AJBC cell line. (**C**) BLAST analysis of the obtained COI sequence against the NCBI nucleotide database.

A fragment of the mitochondrial cytochrome c oxidase subunit I (COI) gene was amplified from AJBC cells at passage 30 to verify the species origin of the established cell line. A single 678-bp PCR product with the expected size was obtained and subsequently sequenced (Figure 3A,B). The obtained COI sequence showed more than 99% sequence identity with the corresponding *Anguilla japonica* COI sequence deposited in the NCBI database (Figure 3C).

### 3.5. Chromosome Analysis

**Figure 4:**
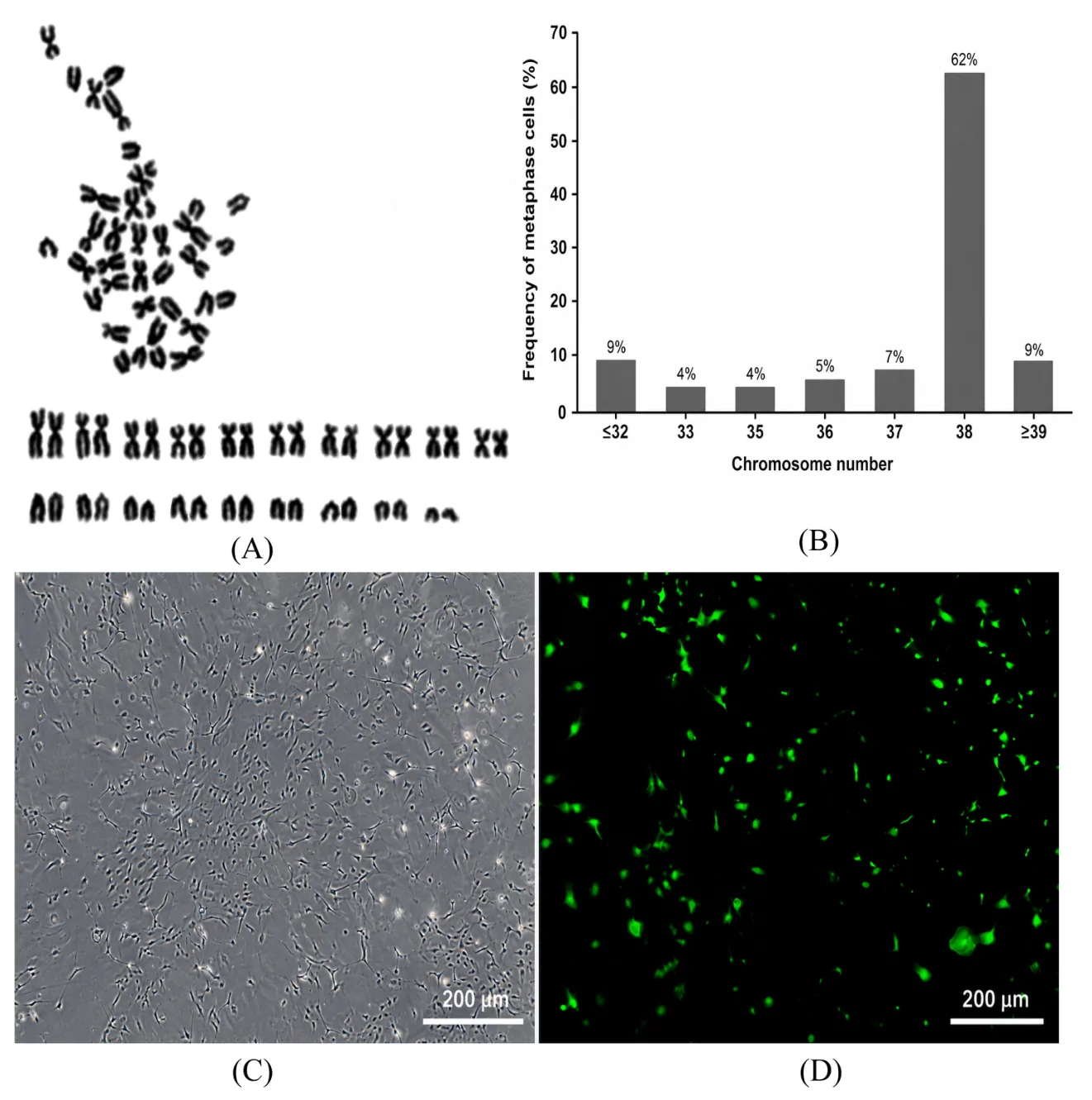
Cytogenetic characterization and transfection capability of the brain-derived AJBC cell line from Japanese eel (*Anguilla japonica*). (**A**) Representative metaphase chromosome spread and diploid karyotype of AJBC cells at passage 30. (**B**) Chromosome number distribution of AJBC cells analyzed from 100 metaphase spreads, showing a consistent chromosome number of 38 (2n = 38). (**C**) Bright-field image of AJBC cells 48 h after GFP plasmid transfection. (**D**) Corresponding fluorescence image showing GFP expression in AJBC cells 48 h after transfection.

Chromosome analysis was performed using AJBC cells at passage 30. Well-spread metaphase chromosomes were successfully obtained after colchicine treatment, hypotonic swelling, fixation, and Giemsa staining (Figure 4A). A total of 100 well--spread metaphase cells were examined for chromosome numbers. The chromosome counts showed that most metaphase cells contained 38 chromosomes, while a small proportion of cells exhibited chromosome numbers deviating from 38 (Figure 4B). The predominant chromosome number of 38 is consistent with the reported diploid chromosome number of *Anguilla japonica* (2n = 38).

### 3.6. Cell Transfection and GFP Expression

AJBC cells were transiently transfected with a GFP-expressing plasmid using a liposome-based transfection method. At 48 h post-transfection, the cells showed normal morphology under bright-field microscopy (Figure 4C), while clear green fluorescence was detected in the corresponding cells under fluorescence microscopy (Figure 4D). Quantification of GFP-positive cells from randomly selected fields revealed a transfection efficiency of approximately 35%. The negative control showed that the cells maintained normal morphology and viability without substantial cell death, suggesting that the transfection reagent itself did not induce severe cytotoxic effects under the experimental conditions (Supplementary Figure S2A,B).

### 3.7. Molecular Characterization of AJBC Cells

**Figure 5:**
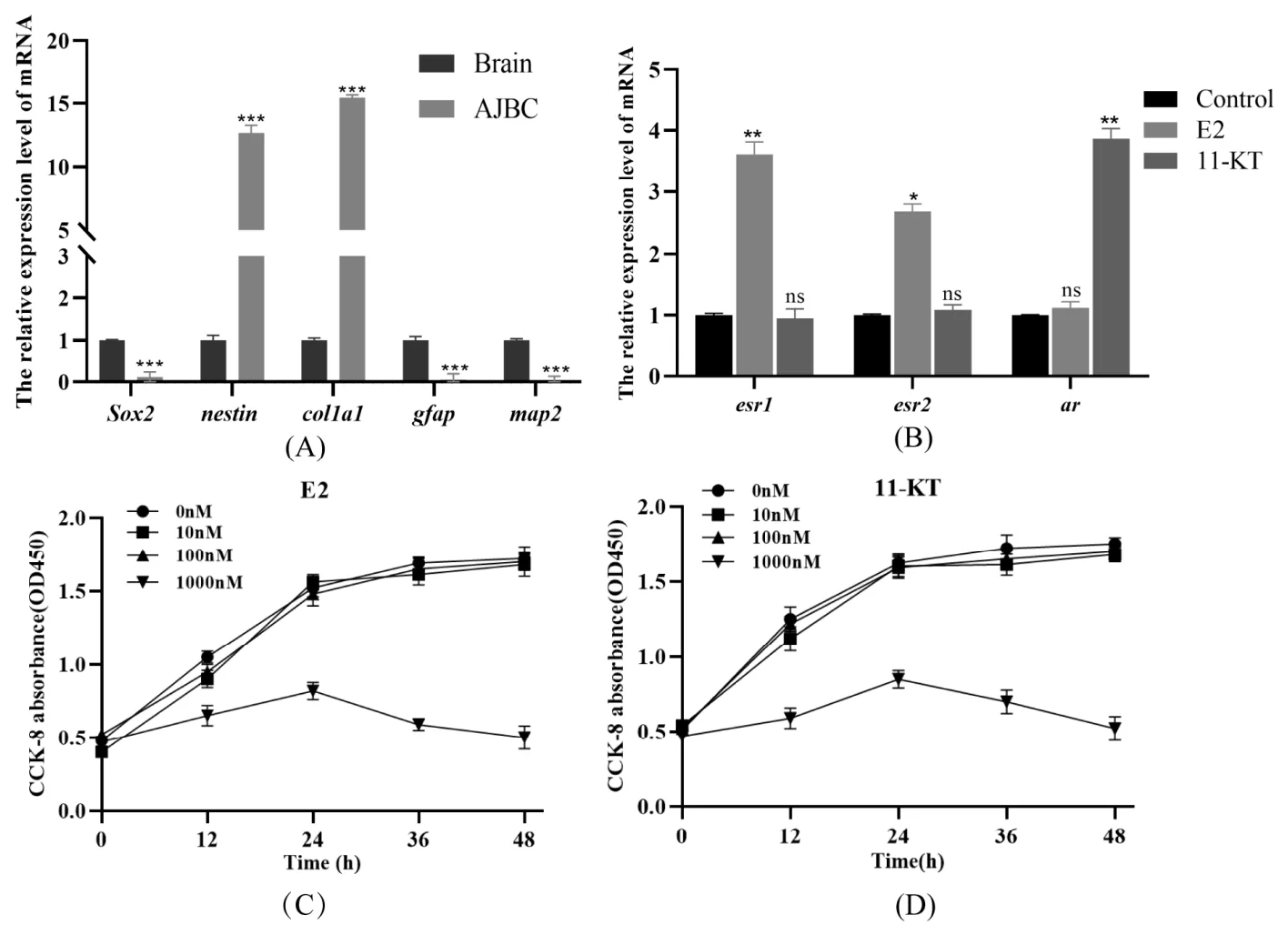
Molecular characterization and sex steroid hormone responses of the brain-derived AJBC cell line from Japanese eel (*Anguilla japonica*). (**A**) Relative mRNA expression levels of neural- and stromal-associated marker genes in AJBC cells compared with brain tissue. (**B**) Expression profiles of sex steroid receptor genes in AJBC cells following E2 or 11−KT treatment. (**C**) Effects of different concentrations of E2 on AJBC cell proliferation at different incubation times measured using the CCK–8 assay. (**D**) Effects of different concentrations of 11−KT on AJBC cell proliferation at different incubation times measured using the CCK-8 assay. Data are presented as mean ± SD (*n* = 3). Statistical significance was determined using two-way ANOVA followed by Tukey’s multiple comparison test. (* *p* < 0.05, ** *p* < 0.01, *** *p* < 0.001; ns, not significant).

The expression levels of neural-associated marker genes were compared between AJBC cells and Japanese eel brain tissues. Compared with brain tissues, AJBC cells exhibited significantly lower expression levels of *sox2*, *map2*, and *gfap*. Notably, the expression levels of *map2* and *gfap* were extremely low in AJBC cells. In contrast, *nestin* and *col1a1* showed significantly higher expression levels in AJBC cells than in brain tissues (Figure 5A).

### 3.8. E2 and 11−KT Hormone Treatment of AJBC Cells

AJBC cells were treated with 0, 10, 100, and 1000 nM E2 or 11−KT for 12, 24, 36, and 48 h, and cell viability was subsequently assessed using the CCK–8 assay. AJBC cells showed relatively stable viability following treatment with 10 and 100 nM E2 or 11−KT throughout the experimental period. In contrast, 1000 nM E2 or 11−KT significantly reduced cell viability compared with the vehicle control, with the reduction becoming more pronounced with prolonged treatment (Figure 5C,D).

### 3.9. Expression Analysis of Sex Steroid Hormone Receptors in AJBC Cells

The expression profiles of estrogen and androgen receptors were analyzed in AJBC cells following E2 and 11−KT treatment. E2 treatment significantly increased the expression levels of *esr1* and *esr2* compared with the control group. In contrast, 11−KT treatment significantly upregulated the expression of *ar*, while no significant changes were observed in estrogen receptor gene expression following 11−KT treatment (Figure 5B).

### 3.10. Transcriptomic Analysis of AJBC Cells to E2 and 11−KT Treatment

**Figure 6:**
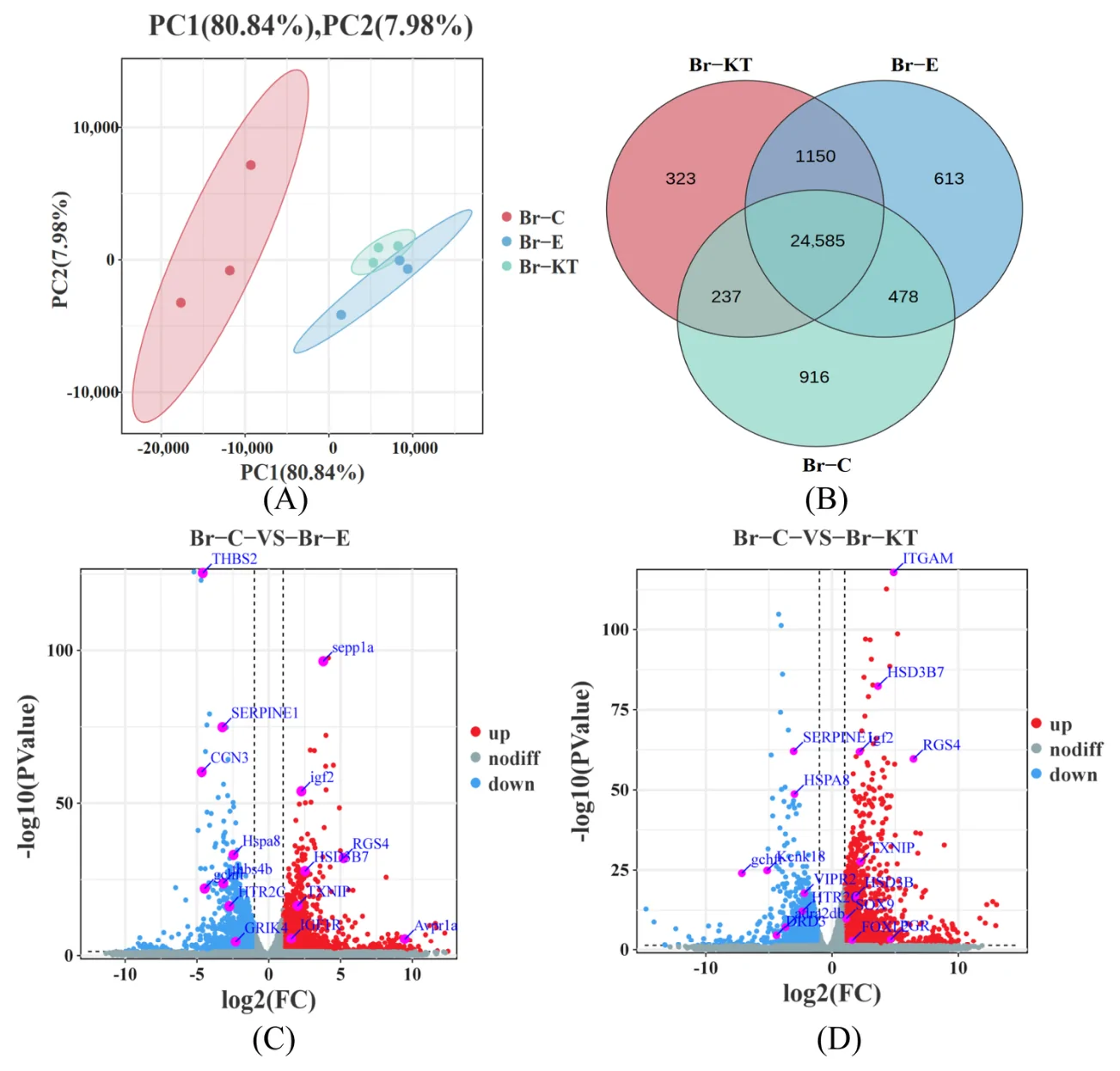
Transcriptomic responses of the brain-derived AJBC cell line from Japanese eel (*Anguilla japonica*) to E2 and 11−KT stimulation. (**A**) Principal component analysis (PCA) of AJBC cells from the control (Br−C), E2-treated (Br−E), and 11−KT-treated (Br−KT) groups. PC1 and PC2 explained 80.84% and 7.98% of the total variance, respectively. (**B**) Venn diagram showing the numbers of shared and group−specific expressed genes among the Br−C, Br−E, and Br−-KT groups. (**C**) Volcano plot showing differentially expressed genes (DEGs) between the Br−C and Br-E groups. (**D**) Volcano plot showing DEGs between the Br–C and Br−KT groups. The vertical dashed lines represent the threshold of |log_2_(fold change)| = 1 (corresponding to a 2-fold change), and the horizontal dashed line indicates the significance threshold of *p* = 0.05. Red and blue dots indicate upregulated and downregulated genes, respectively, and representative DEGs are highlighted.

Transcriptome sequencing was performed to examine gene expression profiles in AJBC cells following treatment with E2 and 11−KT. Principal component analysis (PCA) showed that biological replicates within each group clustered closely together, while the Br–E and Br–KT groups were separated from the Br–C control group (Figure 6A). The Venn diagram showed the numbers of shared and group-specific expressed genes among the Br-C, Br-E, and Br-KT groups (Figure 6B). Differential expression analysis identified a total of 3212 differentially expressed genes (DEGs) between the Br–C and Br-E groups, including 1826 upregulated and 1386 downregulated genes. In comparison, 3522 DEGs were identified between the Br–C and Br–KT groups, including 1974 upregulated and 1548 downregulated genes (Figure 6C,D). Volcano plots showed the distribution of differentially expressed genes in response to E2 and 11−KT treatment, with representative genes showing marked expression changes highlighted (Figure 6C,D).

**Figure 7:**
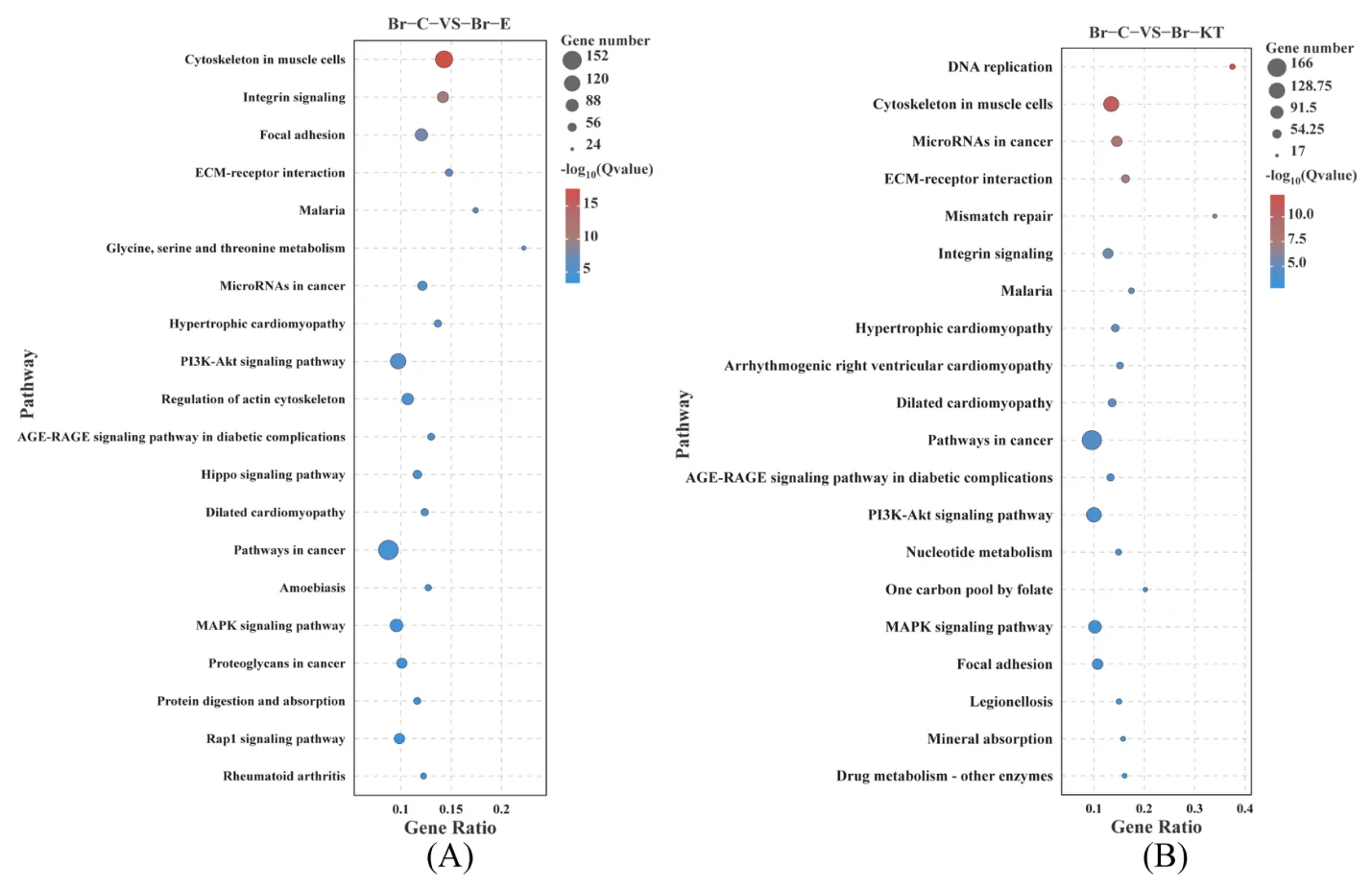
KEGG pathway enrichment analysis of differentially expressed genes in the brain−derived AJBC cell line from Japanese eel (*Anguilla japonica*) following E2 and 11−KT stimulation. (**A**) Top enriched KEGG pathways identified from DEGs between the Br−C and Br−-E groups. (**B**) Top enriched KEGG pathways identified from DEGs between the Br-C and Br−KT groups. The *x*−axis represents the gene ratio, bubble size indicates the number of enriched genes, and bubble color represents enrichment significance based on the −log10(Q−-value).

KEGG pathway enrichment analysis was performed to characterize the biological pathways associated with the differentially expressed genes following E2 and 11−KT treatment. As shown in Figure 7A, DEGs in the Br–C–vs–Br–E comparison were significantly enriched in multiple pathways, with prominent enrichment observed in cytoskeleton-related and cell adhesion-associated pathways, including Cytoskeleton in muscle cells, Integrin signaling, Focal adhesion, and ECM–receptor interaction. Other significantly enriched pathways included the PI3K–Akt signaling pathway, Regulation of actin cytoskeleton, MAPK signaling pathway, and Hippo signaling pathway.

In the Br-C-vs-Br-KT comparison, DEGs were also significantly enriched in multiple pathways (Figure 7B). Among these, the PI3K-Akt and MAPK signaling pathways showed significant enrichment. Other significantly enriched pathways included DNA replication, Cytoskeleton in muscle cells, MicroRNAs in cancer, ECM–receptor interaction, Mismatch repair, Integrin signaling, Focal adhesion, Nucleotide metabolism, and One carbon pool by folate.

**Figure 8:**
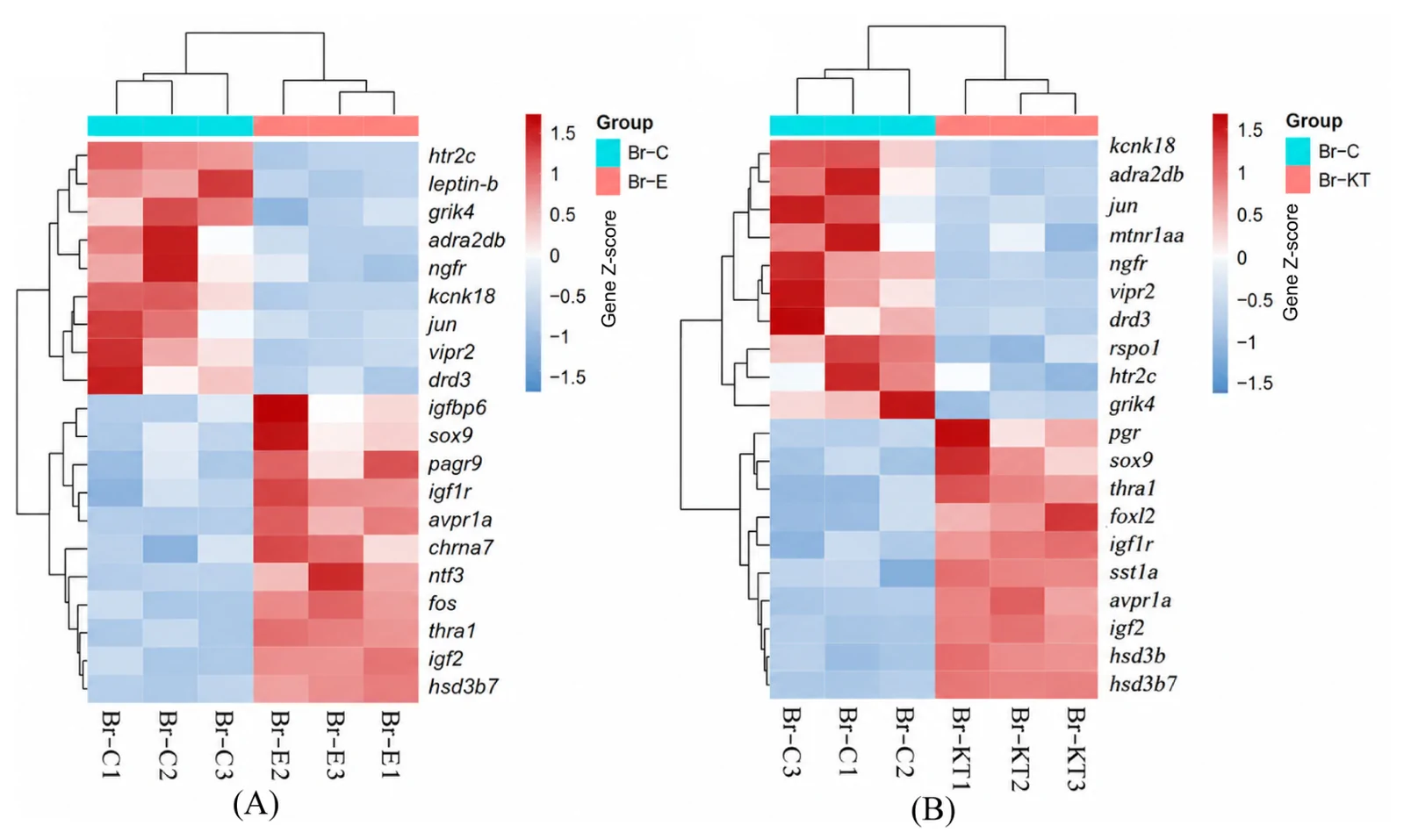
Hierarchical clustering analysis of representative hormone-responsive genes in the brain-derived AJBC cell line from Japanese eel (*Anguilla japonica*) following E2 and 11−KT stimulation. (**A**) Expression patterns of representative genes in the Br−C and Br−E groups. (**B**) Expression patterns of representative genes in the Br−C and Br−KT groups. Each column represents an individual biological replicate, and the color scale indicates normalized relative gene expression levels.

To further characterize the transcriptional responses of AJBC cells to sex steroid hormones, representative differentially expressed genes related to steroid signaling, neuroendocrine regulation, and reproductive processes were selected from the transcriptome dataset for hierarchical clustering analysis. For heatmap visualization, the expression levels of selected genes were standardized using Z-score normalization. As shown in Figure 8, distinct expression patterns were observed between the hormone-treated and control groups.

In the E2-treated group, genes including *igf2*, *igf1r*, *paqr9*, *thra1*, *ntf3*, *vipr2*, and *ngfr* showed relatively higher expression levels, whereas several other genes exhibited lower expression levels compared with the Br−-C group (Figure 8A). In the 11−KT-treated group, a distinct expression pattern was observed, with genes including *sox9*, *hsd3b7*, *fos*, *jun*, *leptin−B*, *drd3*, and *htr2c* showing differential expression between the Br−KT and Br−C groups (Figure 8B). Notably, the sex−-related transcription factors *sox9* and *foxl2* exhibited altered expression patterns following sex steroid treatment. Overall, the hierarchical clustering analysis revealed distinct transcriptional patterns of representative hormone-responsive genes following E2 and 11−-KT treatment.

### 3.11. Validation of RNA-Seq Results by Quantitative Real-Time PCR

**Figure 9:**
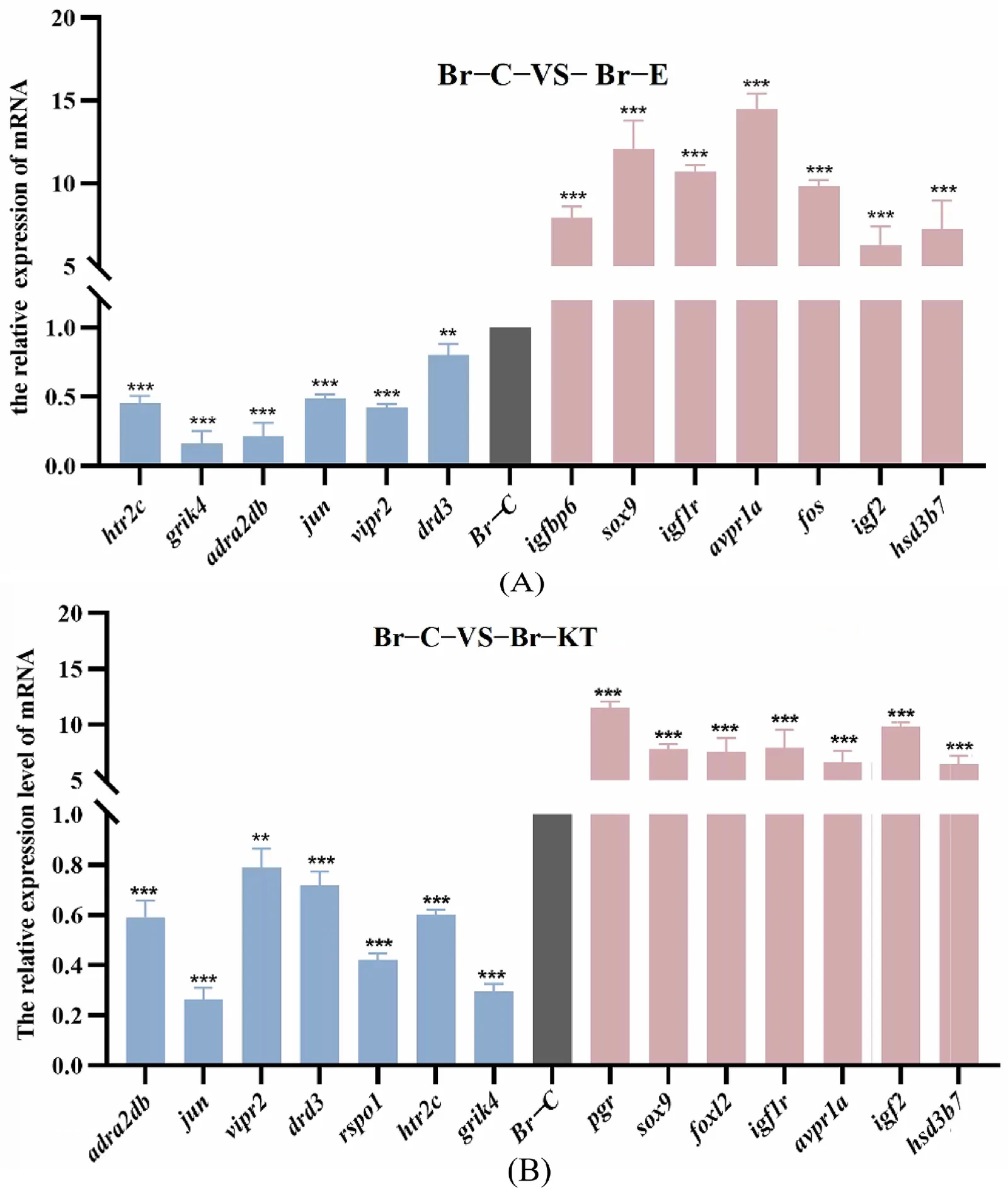
Validation of RNA-seq results by quantitative real-time PCR (qRT−PCR) in the brain-derived AJBC cell line from Japanese eel (*Anguilla japonica*). (**A**) Relative expression levels of representative genes in the Br−C and Br−-E groups following E2 stimulation. (**B**) Relative expression levels of representative genes in the Br-C and Br−KT groups following 11−KT stimulation. Data are presented as mean ± SD (*n* = 3). Asterisks indicate significant differences between hormone-stimulated and control groups (** *p* < 0.01, *** *p* < 0.001).

The expression stability of three candidate reference genes (*18S* rRNA, *β-actin*, and *gapdh*) was first evaluated between control and hormone-treated groups. All three genes showed stable expression patterns, and *β-actin* was selected as the reference gene for qRT-PCR normalization. The amplification efficiency of *β-actin* primers was also validated and showed acceptable performance (Supplementary Figure S3A,B). In the E2−treated group, the expression levels of *htr2c*, *grik4*, *adra2db*, *jun*, *vipr2*, and *drd3* were significantly decreased compared with the control group, whereas *igfbp6*, *sox9*, *igf1r*, *avpr1a*, *fos*, *igf2*, and *hsd3b7* were significantly upregulated following E2 treatment (Figure 9A). In the 11−KT−treated group, *adra2db*, *jun*, *vipr2*, *drd3*, *rspo1*, *htr2c*, and *grik4* were significantly downregulated, while *pgr*, *sox9*, *foxl2*, *igf1r*, *avpr1a*, *igf2*, and *hsd3b7* were significantly upregulated after 11−KT treatment (Figure 9B). The qRT−PCR results showed expression trends consistent with those obtained from the RNA-seq analysis, supporting the reliability of the transcriptomic data and indicating that AJBC cells exhibited distinct transcriptional responses to E2 and 11−KT treatments.

## 4. Discussion

The Japanese eel (*Anguilla japonica*) is an economically important catadromous fish species in East Asia, but its complex reproductive physiology and limited artificial reproduction remain challenges for sustainable aquaculture [27,28]. Stable and experimentally tractable cell models can provide useful tools for investigating cellular and molecular processes in this species [29,30,31,32,33]. In the present study, we established a continuous brain-derived cell line from Japanese eel, designated AJBC, and characterized its basic biological properties. AJBC cells exhibited a relatively uniform fibroblast-like morphology and maintained continuous proliferation during approximately 100 days of culture. Mitochondrial COI sequencing supported the species origin of the cell line, while cytogenetic analysis showed that most metaphase cells contained 38 chromosomes (2n = 38), consistent with the reported diploid chromosome number of Japanese eel. In addition, AJBC cells were successfully recovered after cryopreservation, with post-thaw viability ranging from 75% to 84% across passages P5–P20 and supported transient GFP expression following plasmid transfection. Collectively, these characteristics demonstrate that AJBC cells can be continuously cultured, cryopreserved, recovered, and genetically manipulated, providing a stable and experimentally tractable cell model for further cellular and molecular studies of Japanese eel.

Optimization of culture conditions is important for maintaining stable proliferation of newly established fish cell lines, as serum requirements and temperature preferences vary among cell types and species [29,34,35]. In the present study, AJBC cells proliferated under all tested conditions, but showed the highest proliferation in medium containing 20% FBS and at 27 °C. These results indicate that 20% FBS and 27 °C provide favorable conditions for the routine propagation of AJBC cells and establish a stable culture condition for subsequent experiments.

The cellular identity of AJBC cells was further characterized by examining representative marker genes associated with neural and stromal cell populations. Although AJBC cells were established from brain tissue, they exhibited fibroblast-like morphology and showed high expression of *col1a1*, a gene associated with extracellular matrix production and fibroblast–like stromal populations [36]. In contrast, neuronal and glial markers, including *map2* and *gfap* [24,26], as well as the neural progenitor-associated marker *sox2* [26], showed minimal expression in AJBC cells, suggesting that these cells do not exhibit typical molecular characteristics of neuronal or glial populations. The expression of *nestin* indicates that AJBC cells may retain certain neural-associated characteristics [26], which have also been observed in some primary fish brain-derived cultures. Therefore, AJBC cells may represent a brain-derived stromal–like cell model with neural-associated features rather than a defined neuronal or glial cell type.

Sex steroid hormones are important regulators of reproductive and neuroendocrine functions in teleosts and can act directly on the brain through steroid-responsive signaling pathways [37,38,39,40]. In the present study, transcriptome sequencing revealed extensive transcriptional changes in AJBC cells following E2 and 11−KT treatment, demonstrating that the established cell line retained a clear steroid-responsive phenotype. Notably, 11−KT treatment resulted in a slightly larger number of differentially expressed genes than E2 treatment, and the two treatments exhibited distinct gene-expression patterns. These results suggest that E2 and 11−KT regulate both shared and hormone-specific transcriptional programs in AJBC cells. Such differential responses are consistent with the distinct signaling properties of estrogenic and androgenic pathways and suggest that AJBC cells can integrate different steroid signals at the cellular level. Importantly, the selected hormone concentration of 100 nM did not cause the marked reduction in cell proliferation observed at 1000 nM in the CCK–8 assay, demonstrating that the transcriptional changes detected at 100 nM were not primarily associated with overt cytotoxicity. Nevertheless, because 20% FBS was retained during hormone treatment to maintain cell growth, the potential contribution of endogenous steroid hormones in serum cannot be completely excluded. Future experiments using steroid-depleted serum will help further distinguish the direct effects of exogenous E2 and 11−KT.

The pathway enrichment analysis revealed both shared and hormone–specific responses to E2 and 11−KT in AJBC cells (Figure 6). Both treatments enriched ECM-receptor interaction, integrin signaling, focal adhesion, PI3K-Akt, and MAPK pathways, suggesting that extracellular matrix interactions and intracellular signaling may represent common cellular responses to steroid treatment. Among these pathways, the PI3K-Akt pathway is particularly relevant because it serves as an important downstream signaling hub that integrates extracellular signals, including those mediated by steroid hormones and growth factors, to regulate cell survival, proliferation, metabolism, and gene expression [41,42,43]. In teleosts, estrogen- and androgen-related signaling has been linked to IGF–associated PI3K–Akt and ERK signaling, providing a potential connection between steroid treatment and the cellular responses observed in AJBC cells [41]. Consistent with this possibility, the differential expression of *igf1r* and *igf2* observed in the present study further suggests that IGF-related signaling may contribute to the hormone-responsive transcriptional changes in AJBC cells [42,44]. In contrast, E2 additionally enriched the Hippo signaling pathway, whereas 11−KT was associated with DNA replication and nucleotide metabolism, indicating hormone-specific effects on cellular processes [45]. Thus, the shared enrichment of PI3K–Akt and MAPK pathways, together with the hormone–specific pathway responses, may contribute to the distinct transcriptional profiles induced by E2 and 11−KT. However, KEGG enrichment reflects associations among differentially expressed genes rather than direct pathway activation, and the involvement of these signaling pathways requires further functional validation.

The differential expression of individual genes further illustrates the distinct biological responses of AJBC cells to E2 and 11−KT (Figure 7). Several hormone-responsive genes were associated with neuroendocrine signaling, including *vipr2* and *avpr1a*, suggesting that steroid treatment may influence neuropeptide–-related regulatory processes in AJBC cells, consistent with the interaction between sex steroid signaling and neuroendocrine regulation in the teleost brain [46,47,48,49]. The differential expression of *igf1r* and *igf2* is also notable because IGF-related signaling can interact with PI3K-Akt and MAPK pathways and has been implicated in steroid-responsive cellular processes in fish [42,44], providing a potential molecular link between the gene–level responses and pathway enrichment observed in this study. Genes associated with sex-related and reproductive processes, particularly *sox9*, also showed hormone-responsive expression patterns. In Japanese eel, *sox9a* is associated with testicular differentiation and increases during male gonadal differentiation [28]. However, the absence of significant responses of canonical gonadal differentiation genes such as *dmrt1* and *cyp19a1* indicates that the altered expression of sox9 should not be interpreted as evidence of gonadal differentiation. Rather, these findings suggest that AJBC cells retain selected molecular components linking steroid signaling with reproductive and sex-related regulation. The hormone-specific responses of additional genes, including *htr2c* [50,51], *adra2db* [52], *jun* [53], *drd3* [54], *grik4* [55], *hsd3b7* [56], *chrna7* [57], *ntf3* [58], *fos* [59], and *paqr9* [60], further indicate that steroid treatment affects multiple neural, signaling, and cellular regulatory processes. Collectively, these hormone-responsive genes provide candidate molecular targets for further investigating how steroid signals are integrated in brain-derived cells of Japanese eel.

Importantly, qRT–PCR analysis of representative genes showed expression trends consistent with the RNA-seq results, supporting the reliability of the transcriptomic data. Taken together, AJBC cells exhibited clear and distinct responses to E2 and 11−KT treatment, involving multiple signaling pathways and hormone-responsive genes. Although AJBC cells do not directly represent gonadal differentiation processes, their stable growth characteristics and steroid-responsive properties provide a useful in vitro model for investigating sex steroid-related molecular mechanisms in Japanese eel.

## 5. Conclusions

In this study, we established and characterized a stable brain-derived cell line from Japanese eel and demonstrated its responsiveness to E2 and 11−KT treatment. Transcriptomic analysis revealed both shared and hormone-specific responses, identifying candidate signaling pathways and genes involved in steroid-responsive cellular processes. Although further functional validation is required, AJBC cells provide a useful in vitro model for investigating sex steroid-responsive mechanisms and related neuroendocrine regulation in Japanese eel.

## 6. Patents

A patent application resulting from this work has been filed with the China National Intellectual Property Administration (CNIPA) for the establishment and application of a Japanese eel brain-derived cell line. The invention is titled “A Japanese eel brain tissue cell line AJ-B, preparation method and applications” (Chinese patent application No. 2026111841959; filed on 6 August 2026). The patent application is currently under examination.

## Figures and Tables

**Table 1:** Quantitative real-time PCR primer sequences used for gene expression analysis in Japanese eel (*Anguilla japonica*) cells.

Primer Name	Primer Sequences 5′-3′
*β-actin-F*	CCTTCTACAATGAGCTGCGTGTG
*β-actin-R*	GGTGTTGAAGGTCTCAAACATGATC
*adra2db-F*	GGATCTGACCTCGCAGACTAC
*adra2db-R*	CTGGTGAACACGGCGACAAC
*avpr1a-F*	CCTGTCAGCGTACTCCTCTAC
*avpr1a-R*	CAAGGTGATGCTCAGAACTGCG
*chrna7-F*	GCTATCGGTGGTCTTGCTTCTC
*chrna7-R*	TCTCATCCACATCCATAATCTGCG
*drd3-F*	ATGGACATCGCCTTTGGAAAG
*drd3-R*	CAGAAGCGCGTTATTAGCGGTC
*fos* *-F*	GATGTACACAGGCTTCGGTTCAG
*fos* *-R*	CAGTCGGGATGAAAGATGTGC
*foxl2-F*	CCTCGACATGGCATGCGAAG
*foxl2-R*	GCTCATGAAACTAGACTGCAGG
*grik4-F*	GAGAACCACCACCAGCCTAC
*grik4-R*	CTCAGGAGCTCGAAGATGTCC
*hsd3b7-F*	GTCTACGCAGGTAATGTAGCCTG
*hsd3b7-R*	CTTCAGGAGCCAGTGCAGCAG
*htr2c-F*	CATGGACCGCAACAGCAACTG
*htr2c-R*	TCATACGCAGCTGGTGAGCTC
*igf1r-F*	ATGAGGTCTGGAGGAGCAAAGG
*igf1r-R*	GTCTTGTCGCCGATGAGCAG
*igfbp6-F*	CAGCAGGTCCATACCCTTCAC
*igfbp6-R*	TGCTCGTCCACGCACCAG
*igf2-F*	CTCTCTGTCACACCTGCGTAAG
*igf2-R*	GAAGCCTCTGTCTTCACATACG
*jun* *-F*	CTCGCTGAATGCCGCTTTCTC
*jun* *-R*	CAGCTTTAGCAGTCCGACATC
*pgr* *-F*	GGAACATGTGACGAGGATTCAG
*pgr* *-R*	GATTCTGGAGACGAGGTCGG
*rspo1-F*	CATACTGCTGGAGCGCAACG
*rspo1-R*	CTGTGGTGCCGTTAGTGGTG
*sox9-F*	GCATGTCAGAGGATTCTGCAGAG
*sox9-R*	GTCCAGTCGTAGCCCTTCAAC
*vipr2-F*	GATGGTCAGCAATCCTTGTCCAG
*vipr2-R*	GTGTCCCAGTGTGTACAGAGTC
*ar* *-F*	GCCTTCAAGTGGCATTTCAGAGTC
*ar* *-R*	CGTCTTCGTTTGTCCCAGTGTC
*esr1-F*	ATGGCAAGGCCGTCTTCTGC
*esr1-R*	GTAGCTGATCGCCACGGCTC
*esr2-F*	CGACTCCGCAAGTGCTATGAAG
*esr2-R*	GTTGATTAACTGCTCCGGCGTG
*gfap* *-F*	CCAGCGAGAAGCTCCAGCTG
*gfap* *-R*	GGAGCTCCTCCTGGTAGACC
*map2-F*	CTGAGGAGGAAGAGGAGACTGC
*map2-R*	ACACTTCTCCAACCCTGCCTC
*nestin* *-F*	ATGGAGGTCCCAGTGACTCG
*nestin* *-R*	CTTTCCTCAGTTGGCCCTCCAG
*sox2-F*	GCACCTCAGCCCAATTCTGG
*sox2-R*	CTTCGCCTCGTCGATGAAAGG
*col1a1-F*	CAACTGGTGAGACTGGCAAGC
*col1a1-R*	CTGCGTCACCCTTAGCACCATC
*18s-F*	AACGAGGATCCATTGGAGGGC
*18S-R*	GGACACTCAGCTAAGAGCATCG
*gapdh* *-F*	CGTCATCTCCGTCTTCCAGTG
*gapdh* *-R*	GTCCTCGTTCACGCCCATG

**Table 2:** Post-thaw cell viability of the brain-derived AJBC cell line from Japanese eel (*Anguilla japonica*).

Cryopreserved Cell Passage	Viability After Thawing
P5	75%
P10	81%
P15	77%
P20	84%

## Data Availability

The RNA-seq data generated in this study have been deposited in the NCBI Sequence Read Archive (SRA) under BioProject accession number PRJNA1526325 and are publicly available at https://dataview.ncbi.nlm.nih.gov/object/PRJNA1526325 accessed on 15 September 2025.

## Supplementary Materials

The following supporting information can be downloaded at: https://www.mdpi.com/article/10.3390/ani16182951/s1, Figure S1: Histological section of Japanese eel (*Anguilla japonica*) gonad. Hematoxylin and eosin (H&E) staining, showing oocytes at different developmental stages. Scale bar: 100 μm.; Figure S2: Brightfield and GFP fluorescence observation of negative control in Japanese eel (*Anguilla japonica*) brain cell line. (**A**) Brightfield image showing adherent Japanese eel brain cells. (**B**) GFP channel: no green fluorescent signal was observed, indicating no GFP expression in negative control cells; Figure S3: qPCR reference gene detection in Japanese eel (*Anguilla japonica*) brain cell line. (**A**) Ct values of *β-actin*, *gapdh* and *18S* genes in different treatment groups. (**B**) Amplification standard curves of *β-actin* in control, E_2_−treated, and 11-KT-treated groups.
