# Supplementary material for: Establishment and Characterization of a Brain-Derived Cell Line from Japanese Eel (Anguilla Japonica) and Transcriptomic Analysis of Its Responses to Sex Steroid Hormones
Source: Animals (Basel). 2026 Sep 19;16(18):2951. doi: 10.3390/ani16182951 (PMC13603464; doi:10.3390/ani16182951)
Supplement: Supplementary file 1 [file animals-16-02951-s001.zip › animals-4539641-supplementary.pdf]

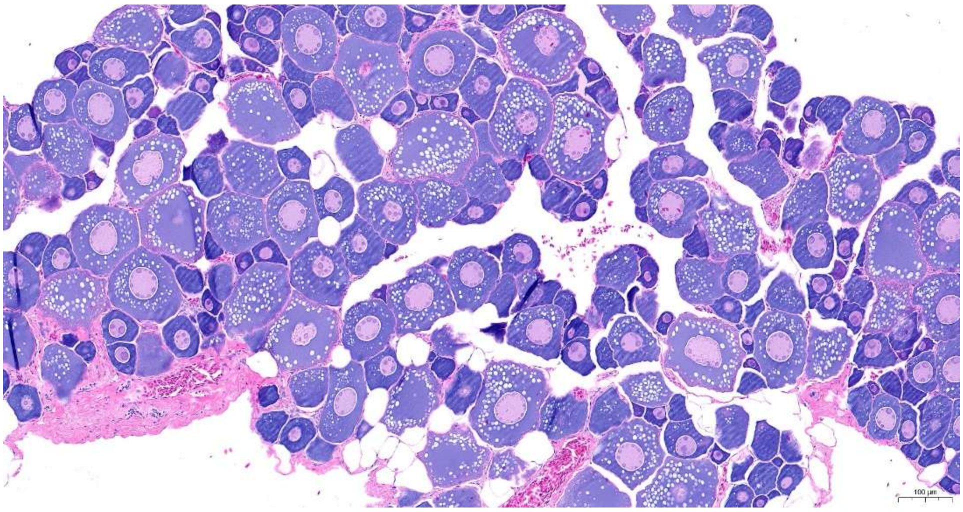

Figure S1. Histological section of Japanese eel (*Anguilla japonica*) gonad. Hematoxylin and eosin (H&E) staining, showing oocytes at different developmental stages. Scale bar: 100 μm.

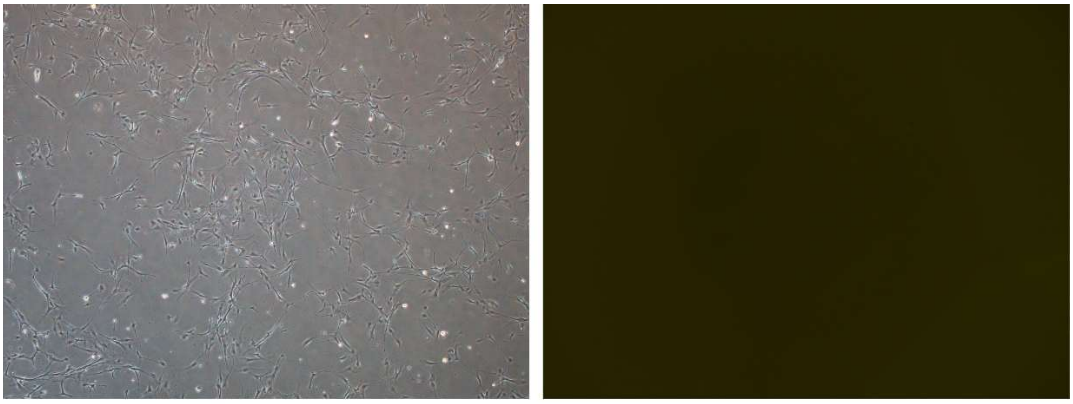

(A) (B)

Figure S2. Brightfield and GFP fluorescence observation of negative control in Japanese eel (*Anguilla japonica*) brain cell line. (A) Brightfield image showing adherent Japanese eel brain cells. (B) GFP channel: no green fluorescent signal was observed, indicating no GFP expression in negative control cells.

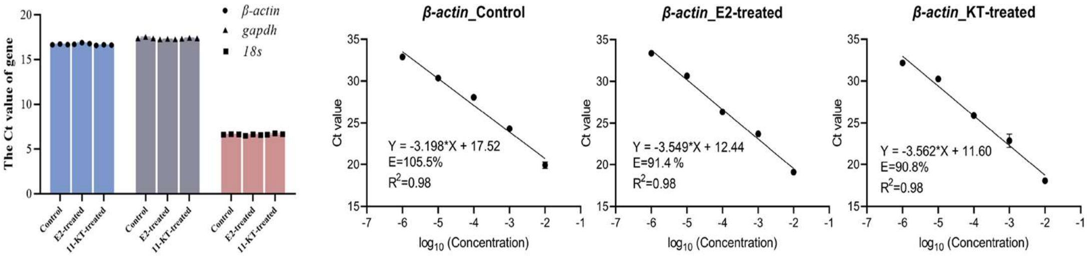

(A) (B)

Figure S3. qPCR reference gene detection in Japanese eel (*Anguilla japonica*) brain cell line. (A) Ct values of *β-actin*, *gapdh* and *18s* genes in different treatment groups. (B) Amplification standard curves of β-actin in control, E_2-treated, and 11-KT-treated groups.
